# Multi-layer robotic controller for enhancing the safety of mobile robot navigation in human-centered indoor environments

**DOI:** 10.3389/frobt.2025.1629931

**Published:** 2025-07-31

**Authors:** Karameldeen Omer, Andrea Monteriù

**Affiliations:** ^1^ Department of Information Engineering, Università Politecnica delle Marche, Ancona, Italy; ^2^ Department of Mechanical Engineering, University of Khartoum, Khartoum, Sudan

**Keywords:** multi-tier robot controller, autonomous navigation, human-in-the-loop, semantic mapping, virtual barriers, robot safety, building information models (BIM), robotic digital twins

## Abstract

This research proposes a multi-layer navigation system for indoor mobile robots when they share space with vulnerable individuals. The primary objectives are increasing or maintaining safety measures and curtailing operational costs, emphasizing reducing reliance on intricate sensor technologies and computational resources. The developed system employs a three-tiered control approach, with each layer playing a pivotal role in the navigation process. The “online” control layer integrates a human-in-the-loop strategy, where the human operator detects missing obstacles or approaching danger through a user interface and sends a trigger to the robot’s controller. This trigger enables the system to estimate the coordinates of the danger and update the robot’s navigation path in real time, minimizing reliance on complex sensor systems. The “semi-online” control layer generates dynamic virtual barriers to restrict the robot’s navigation in specific areas during specific times. This ensures the robot avoids hazardous zones that could pose temporary risks to the human or robot. For example, areas with temporary obstructions or potential danger, such as kids’ play zones or during cleaning, are temporarily restricted from the robot’s path, ensuring safe navigation without relying solely on real-time sensor data. The “offline” control layer centers around the use of semantic information to control the robot’s behavior according to user-defined space management and safety requirements. By leveraging Building Information Models (BIM) as digital twins, this layer combines semantic and geometric data to comprehensively understand the environment. It enables the robot to navigate according to precise user requirements, utilizing the semantic context for path planning and behavior control. This layer obviates the need for a real-time sensor mapping process, making the system more efficient and adaptable to user needs. This research represents a significant step forward in enhancing the navigational capabilities of robots within human-centric indoor environments, with a core focus on safety, adaptability, and cost-effectiveness.

## 1 Introduction

### 1.1 Background and safety in indoor mobile robot navigation

The past few decades have witnessed a remarkable boom in the integration of robots into various sectors De Backer et al. (2018). This trend is particularly evident in the deployment of autonomous mobile robots (AMRs) in indoor environments and environmentally assisted living (AAL) applications, driven by significant technological advances and the increasing demand for automation. Robots are now utilized across various industries, including smart homes, healthcare, hospitality, retail, logistics, and education, transforming how tasks are performed in these diverse environments Ray (2016).

Originally, mobile robots were mainly employed in industrial settings where tasks were repetitive and performed under structured and predictable conditions Pedersen et al. (2016). However, as technology has progressed, the scope of these robots’ applications has expanded to include dynamic, unstructured environments such as homes, hospitals, and public spaces. In these environments, robots are expected to operate autonomously, navigate complex spaces, and interact safely with humans and objects.

Historically, navigation in such environments relied heavily on advanced sensor systems, such as LiDAR, ultrasonic sensors, 3D laser scanners, and cameras Liu et al. (2024). These sensors gather extensive environmental data, which is then processed to map surroundings Alatise and Hancke (2020), detect obstacles, and plan the robot’s path. While effective, this approach requires significant computational resources, which adds to costs and limits scalability.

In environments where vulnerable individuals, such as the elderly or those with disabilities, reside, the reliance on such sensor systems presents additional challenges. These environments demand robots that can navigate without posing risks to individuals with limited mobility or other impairments Marques et al. (2019). Furthermore, the cost-effectiveness of deploying and maintaining robotic systems is crucial, given the often limited budgets of public health and social care institutions Tan and Taeihagh (2021).

Thus, the development of automated navigation systems for indoor environments faces a dual challenge Loganathan and Ahmad (2023). On one hand, there is a need to maintain high levels of operational efficiency and autonomy to ensure robots can perform tasks effectively Ciabattoni et al. (2021); Alatise and Hancke (2020). On the other hand, the limitations of heavy reliance on sensor technologies and computational resources must be addressed. Striking a balance between efficiency, safety, and cost-effectiveness is critical to the widespread deployment of these technologies.

In response to these challenges, recent research has shifted towards alternative navigation strategies Ton et al. (2018). These strategies reduce reliance on real-time sensor data, leverage pre-existing environmental information, and incorporate human input to improve navigation decisions Nunes et al. (2015). The aim is to develop robots that are not only efficient and autonomous but also safe and cost-effective for deployment in human-centered environments.

Furthermore, ensuring safety in indoor mobile robot navigation is paramount, particularly as robots increasingly operate in shared, dynamic environments. Multi-Robot Systems (MRSs) are expected to work autonomously and collaborate with humans to complete complex tasks, making them both safety-critical and mission-critical systems Rizk et al. (2019). Despite advancements in robotics, many essential capabilities for reliable operation in unstructured indoor settings remain insufficient, hindering the widespread deployment of robots in real-world scenarios.

A significant aspect of safety in these systems is human-robot interaction (HRI). Studies show that factors like a robot’s appearance, speed, and approach direction impact how safe people perceive the robot to be. Visual cues, such as floor projections and turn indicators, along with haptic feedback systems, can enhance the robot’s predictability and foster trust in shared spaces Rubagotti et al. (2022); Cardoso et al. (2021).

Additionally, the refinement of safety standards, such as ISO/DIS 13482, plays a vital role in guiding the development of safe robotic systems. These updates provide performance requirements, speed limits, and emergency stopping protocols, although they often do not address the nuanced safety challenges in public and semi-public indoor spaces Rose (2020).

Advances in perception and sensor systems, particularly when combining LiDAR, cameras, radar, and ultrasonic sensors, have significantly improved robot safety by enhancing environmental understanding and obstacle detection Yang et al. (2025); Zhang Y. et al. (2024). Additionally, AI-driven approaches enable robots to refine their navigation strategies over time, learning from human presence and adapting to dynamic indoor conditions Zhang Q. et al. (2024).

A holistic approach to safety extends beyond real-time navigation and includes considerations such as payload stability, tipping prevention, surface interaction, and effective emergency planning. These factors contribute to a more robust and versatile system for diverse indoor applications Tan et al. (2016).

Despite these advancements, there remain several gaps in ensuring safety for indoor mobile robots. A review of 58 recent works highlights key deficiencies, particularly the underutilization of knowledge sharing among robots. Few studies explore centralized or cooperative mechanisms, limiting the resilience of robot systems in dynamic environments. The lack of dedicated safety layers, often embedded within functional code, reduces clarity and complicates system verification and maintenance. Moreover, real-time adaptability remains limited, and most research focuses on homogeneous robot teams, with managing heterogeneous systems largely unexplored Bozhinoski et al. (2019).

In conclusion, the integration of mobile robots into indoor spaces presents a dynamic and evolving challenge. As these robots expand into more sensitive and unregulated environments, the need for innovative navigation solutions that balance operational efficiency with safety, cost-effectiveness, and human-robot interaction becomes increasingly clear. Efforts to address these challenges will be crucial in ensuring that the deployment of autonomous robots in human-centric spaces is both safe and effective.

### 1.2 Motivation

The rapid advancement in robotics, particularly in the development of autonomous mobile robots (AMRs) for indoor navigation, has brought forth significant changes across various sectors. However, this progress also presents challenges that need addressing, with a primary focus on enhancing safety, reducing costs, and catering to the needs of environments with vulnerable populations.

Safety is a critical concern, especially in indoor settings where robots interact closely with humans. Advanced sensing technologies used in these robots improve environmental awareness and decision-making, but also add complexity and raise safety risks. Malfunctions or errors can lead to incidents, particularly in densely populated or confined spaces. Thus, developing a navigation system that enhances safety standards is crucial, especially where minor faults can have serious consequences.

Cost reduction is another vital aspect, as current robotic systems, with their sophisticated sensing technologies and computational algorithms, incur high initial and maintenance costs. This limits their accessibility and scalability, particularly for smaller operations or those with limited budgets. Achieving cost-effectiveness without compromising efficiency is essential for wider adoption and further progress in the field.

### 1.3 Problem statement

**FIGURE 1:**
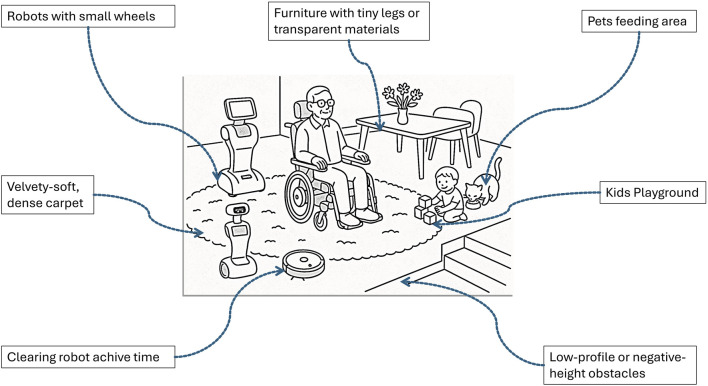
Use the Schematic of a shared indoor environment with humans, robots, and pets. The scene illustrates common navigation challenges that are difficult for typical robot sensors to detect and interpret reliably.

The primary challenge is to enhance safety in indoor navigation, both for the robot and users, while minimizing reliance on advanced sensors without compromising performance. Moreover, the need for solving the critical challenges posed by negative and undetected obstacles,as illustrated in Figure 1, including stairs, ramps, transparent materials, and other problematic elements that traditional sensors often fail to detect effectively as tiny legs or short objects Trautmann et al. (2023).

## 2 Materials and methods

### 2.1 Proposed approach

Building on the identified gaps in the current state of the art regarding safety in indoor mobile robot navigation, this thesis proposes a multi-layered artificial control architecture designed to enhance robot behavior in human-shared environments. The goal is to enable robots to behave in ways that are predictable, interpretable, and manageable by humans, without compromising the robot’s autonomy.

The proposed control system can be integrated with most existing mobile robots, regardless of their hardware limitations. It is designed to emulate typical human indoor behaviors, allowing for intuitive co-existence between humans and robots. By minimizing sensor dependencies and reducing computational overhead, the system enables even low-cost robots with basic navigation capabilities and low-tech sensors to perform comparably to systems using more sophisticated technologies or complex AI models.

Each layer addresses specific behavioral aspects of indoor navigation and collectively ensures that the robot’s actions are aligned with human expectations and safety standards. This approach not only enhances robot predictability and social acceptability but also democratizes access to safe autonomous navigation for a broader range of robotic platforms.

#### 2.1.1 Selecting and designing the layered control architecture

Designing a layered control architecture for safe indoor navigation requires drawing from both engineering principles and natural behavioral models. According to Arkin Arkin (1998), robotic behaviors can be designed through three main strategies: ethologically guided (inspired by animal behaviors), situated activity-based (emerging from specific environmental contexts), and experimentally driven (developed through iterative trials). Our approach blends situated activity-based design and ethologically inspired principles, particularly from natural safety mechanisms observed in animals, alongside practical models from human risk management at home.

From an ethological perspective, animal behavior offers robust, evolutionarily tested strategies for avoiding danger. A particularly relevant example is the safety system in rats, which involves layered responses to perceived threats Prescott and Redgrave (1996). Rats react to danger using a tiered mechanism: immediate stimuli (like a sudden noise or movement) trigger reflexive escape or freezing behaviors; mid-term threats are handled through avoidance strategies or hiding based on prior learning and environmental cues; and long-term safety relies on memory and territorial mapping to avoid dangerous zones. This tiered response system inspired our multi-layered control approach, which mimics such stratified decision-making in robotic systems for indoor navigation Matni et al. (2024).

Translating this into a human domestic context, we observe that people intuitively manage household risks across three levels:

• Immediate Reactions (Online Layer): When someone notices a person approaching immediate danger, like a child nearing a staircase, they often react by shouting or gesturing. This fast, localized response is transient and situation-specific. Our online layer emulates this by enabling the robot to respond instantly to high-risk, real-time events using lightweight sensing and minimal computation.

• Short-Term Contextual Awareness (Semi-Online Layer): In domestic settings, humans frequently impose temporary boundaries, such as avoiding an area being cleaned or where a pet is feeding. These restrictions are valid for short periods and particular locations. Our semi-online layer captures this behavior by enforcing short-term navigational rules that adapt as the environment changes.

• Long-Term Rules and Restrictions (Offline Layer): Certain behaviors, like not entering a room with fragile decorations or keeping away from high-value electronics, are enforced as standing house rules. The offline layer incorporates such persistent constraints into long-term planning and navigation behavior.

**FIGURE 2:**
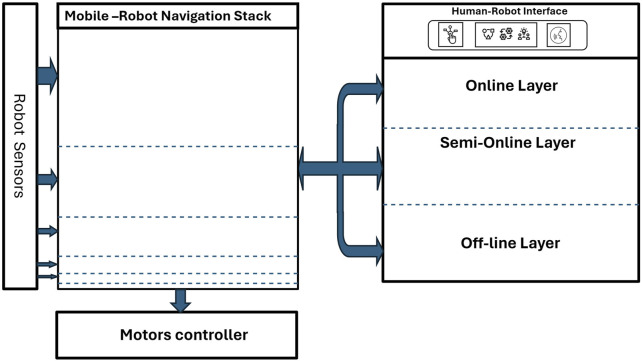
Comprehensive overview of the three-layer control architecture for autonomous robot navigation. The layered controller stack interfaces with the existing robot control system, intercepting and modifying high-level commands to enforce behavior constraints, such as human-centered safety, before relaying them to the unmodified low-level motor controller.

By integrating these biologically and situationally inspired layers, our novel control architecture in Figure 2 ensures that robots behave predictably and safely in shared human environments. It also allows low-complexity robots to perform context-aware navigation without requiring high-end sensors or heavy AI models. This strategy ultimately improves safety, enhances trust, and facilitates smoother human-robot coexistence in indoor settings.

### 2.2 Online (real-time human-in-the-loop)

Human-in-the-loop (HITL) approaches for correcting robots are pivotal in enhancing the accuracy, adaptability, and reliability of robotic systems by integrating human expertise and feedback into the robots’ operational processes. These approaches are especially beneficial in human-robot interaction, making the user an active participant in the control loop. Users can provide immediate feedback or corrections to the robot’s actions, significantly augmenting its capabilities. This becomes crucial in scenarios where safety is paramount, such as in assistive robotics.

In the context of this study, the focus is on realizing a human-in-the-loop approach to empower specific robots, such as mobile robots and smart wheelchairs, by augmenting their artificial sensory sets. The aim is to extend and enhance robotic capabilities for obstacle detection and avoidance by incorporating human feedback mechanisms. For mobile robots, feedback is provided via vocal commands, while in the case of assistive wheelchairs, both a keyboard and a brain-computer interface (BCI) are employed. Furthermore, we adopted a novel method based on a passive BCI that leverages error-related potentials (ErrPs) as a feedback mechanism, as developed by Ferracuti et al. (2022); Omer et al. (2022). This innovative approach not only enhances the robots’ navigational abilities but also ensures a safer and more intuitive interaction for the user. By validating the entire architecture within a simulated robotic environment and analyzing electroencephalography signals from different test subjects, the study demonstrates the effectiveness of HITL in enhancing robot performance through direct human input.

#### 2.2.1 Semi-online control layer: Virtual barriers and doors

The “semi-online” control layer focuses on the establishment of dynamic virtual boundaries. These boundaries are not fixed physical barriers but are instead virtually defined limits within which the robot operates. This layer uses a combination of pre-set rules and real-time environmental data to adjust these boundaries dynamically, ensuring safe navigation. For instance, in a huge living room, the robot would automatically set its path to maintain a safe distance from people and avoid navigating where kids’ play areas or pets’ feeding zones are. This approach strikes a balance between pre-planned navigation and the need for real-time adaptability, ensuring efficient movement while prioritizing safety.

#### 2.2.2 Offline control layer: Utilizing semantic data from Building Information Models

The third and final layer, the “offline” control layer, capitalizes on Building Information Models for path planning and semantic mapping. BIMs are detailed digital representations of the physical and functional characteristics of facilities. By utilizing BIM, the robot can access a comprehensive map of its operating environment, which is represented through labels or categories that carry meaning for humans, such as “kitchen”, “office”, “hallway”, and all the types of furniture and equipment, rather than purely geometric or topological data. This pre-existing data allows for efficient path planning without the need for extensive real-time sensor data processing. The robot can navigate effectively using this detailed environmental blueprint, significantly reducing the computational load and associated costs. This method enables interaction between humans and robots to be more seamless and intuitive.

#### 2.2.3 Robot model and the three-layer controller modeling

This section details the mathematical modeling of the robot, including error computation, PID control, obstacle avoidance, and a layered controller architecture.

### 2.3 Robot model

#### 2.3.1 Kinematic model of a differential-drive robot

The motion of a differential-drive mobile robot is governed by its non-holonomic kinematic equations, assuming no slip and pure rolling:

$$\dot{x}\left(t\right)=v\left(t\right)\cos\left(\theta \left(t\right)\right)$$ (1)

$$\dot{y}\left(t\right)=v\left(t\right)\sin\left(\theta \left(t\right)\right)$$ (2)

$$\dot{\theta }\left(t\right)=\omega \left(t\right)$$ (3)

Where:

• x(t),y(t) : Position of the robot in Cartesian coordinates

• θ(t) : Orientation of the robot

• v(t) : Linear velocity

• ω(t) : Angular velocity

These Equations 1–3 form the foundation for modeling the motion and designing control strategies.

#### 2.3.2 Error computation

The robot’s tracking errors are computed from desired $\left(x_{d}\left(t\right),y_{d}\left(t\right),\theta_{d}\left(t\right)\right)$ and actual (x(t),y(t),θ(t)) states as appears in Equations 4, 5, 7:

$$e_{x}\left(t\right)=x_{d}\left(t\right)-x\left(t\right)$$ (4)

$$e_{y}\left(t\right)=y_{d}\left(t\right)-y\left(t\right)$$ (5)

$$e_{d}\left(t\right)=\sqrt{e_{x}{\left(t\right)}^{2}+e_{y}{\left(t\right)}^{2}}\;\left(\mathrm{DistanceError}\right)$$ (6)

$$e_{\theta }\left(t\right)=\theta_{d}\left(t\right)-\theta \left(t\right)\;\left(\mathrm{OrientationError}\right)$$ (7)

#### 2.3.3 PID controller

• *Linear Velocity:*

$$v\left(t\right)=K_{pv}\cdot e_{d}\left(t\right)+K_{iv}\int e_{d}\left(t\right)dt+K_{dv}\frac{de_{d}\left(t\right)}{dt}$$ (8)

• *Angular Velocity:*

$$\omega \left(t\right)=K_{p\omega }\cdot e_{\theta }\left(t\right)+K_{i\omega }\int e_{\theta }\left(t\right)dt+K_{d\omega }\frac{de_{\theta }\left(t\right)}{dt}$$ (9)

#### 2.3.4 Differential drive kinematics

For a differential drive robot, the required wheel angular velocities are calculated for Equations 8–11:

$$\omega_{l}=\frac{2v_{\text{final}}-\omega_{\text{final}}L}{2R}$$ (10)

$$\omega_{r}=\frac{2v_{\text{final}}+\omega_{\text{final}}L}{2R}$$ (11)

#### 2.3.5 Obstacle avoidance

Obstacle Detection from Sensors:

$$O_{r}=\left\{\left(x_{j},y_{j},z_{j}\right)\right\}$$ (12)

Potential Field Method:

The combined potential field from real and virtual obstacles:

$$\phi \left(x,y,z\right)=\underset{i\in O_{v}}{\sum }\frac{1}{\left\|x-x_{i}\right\|}+\underset{j\in O_{r}}{\sum }\frac{1}{\left\|x-x_{j}\right\|}+\underset{k\in B_{v}}{\sum }\frac{1}{\left\|x-x_{k}\right\|}$$ (13)

Repulsive Force:

Derived from the gradient of the potential field:

$$F_{\text{rep}}\left(x,y,z\right)=-\nabla \phi \left(x,y,z\right)$$ (14)

Adjusted Control Signals:

• *Final Linear Velocity:*

$$v_{\text{final}}\left(t\right)=v\left(t\right)+F_{\text{rep},v}\left(x,y,z\right)$$ (15)

• *Final Angular Velocity:*

$$\omega_{\text{final}}\left(t\right)=\omega \left(t\right)+F_{\text{rep},\omega }\left(x,y,z\right)$$ (16)

#### 2.3.6 Layered controller

Online and Semi-Online Layers:

• Virtual obstacles from BCI feedback:

$$O_{v}=\left\{\left(x_{i},y_{i},z_{i}\right)\right\}$$ (17)

• Virtual barriers if BCI condition f(t)=1 :

$$B_{v}=\left\{\left(x_{k},y_{k},z_{k}\right)\right\}$$ (18)

• Semi-online layer sensor data:

$$B_{s}=\left\{\left(x_{i},y_{i},z_{i}\right)\right\}$$ (19)

Offline Layer: Semantic Path Planning.

• Individual cost component:

$$C_{k}\left(x\right)=\alpha_{k}\cdot f_{k}\left(d_{k}\left(x\right)\right)$$ (20)

• Total cost function:

$$C\left(x,y\right)=\underset{k}{\sum }\alpha_{k}\cdot f_{k}\left(d_{k}\left(x\right)\right)$$ (21)

• Semantic path planning using cost map and obstacles:

$$P\left(t\right)=\text{Path}\left(\text{Mim, Start, Goal},O_{r},C\left(x,y\right)\right)$$ (22)

• Optimization objective:

$$\min\left(\int_{p}\underset{k}{\sum }\alpha_{k}\cdot f_{k}\left(d_{k}\left(x,y\right)\right)ds\right)$$ (23)

**FIGURE 3:**
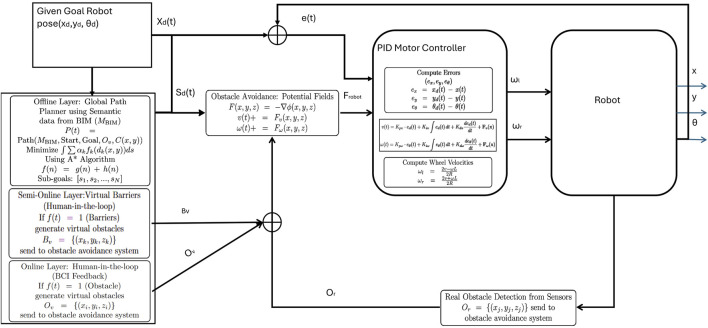
Use the System architecture in the control scheme, illustrating the integrated layered controller that operates above the original robot controller. This controller intercepts and modifies high-level control inputs to embed behaviors such as obstacle avoidance before passing commands to the original, unmodified low-level motor controller.

All the layers described in Equations 12–23 are integrated intothe final control architecture of the robot and the Laird controller, as illustrated in Figure 3.

### 2.4 Experimental setup and simulation framework

This section presents the experimental setup designed to validate our human-in-the-loop navigation strategy for assistive robotics, implemented through a smart wheelchair platform Ciabattoni et al. (2021). The experiments were conducted entirely in simulation, leveraging the Robot Operating System (ROS) Quigley et al. (2009) and the Gazebo 3D simulator to create a realistic and interactive environment for testing path planning, obstacle avoidance, and user interaction mechanisms.

ROS includes all packages for robot control and autonomous navigation. The simulated tests have been realized in Gazebo, where the virtual environment for the simulation of the smart wheelchair movement and the acquisition of all the sensors have been recreated. Gazebo is a 3D simulator developed by the Open-Source Robotics Foundation, with which it is possible to create a 3D scenario with robot obstacles and many other objects. In Gazebo, it is possible to configure the robot as links and joints, and all the equipped sensors are virtualized to be used by ROS packages and nodes. It also uses a physical engine for illumination, gravity, inertia, etc. Gazebo was designed to evaluate algorithms for many applications. It is essential to test the developed robot applications, like error handling, battery life, localization, navigation, and grasping (see Freddi et al., 2021).

#### 2.4.1 Simulation environment

The simulation was carried out in a virtual reconstruction of the corridor at the Department of Information Engineering, Università Politecnica delle Marche. The environment includes structural elements such as walls, virtual holes in the floor, and glass partitions, facilitating the evaluation of the robot’s capabilities under complex and varied conditions. A total of 10 floor holes, each with a diameter of 20 cm and spaced 3 m apart, were introduced to simulate hazardous terrain that is undetectable by onboard sensors.

**FIGURE 4:**
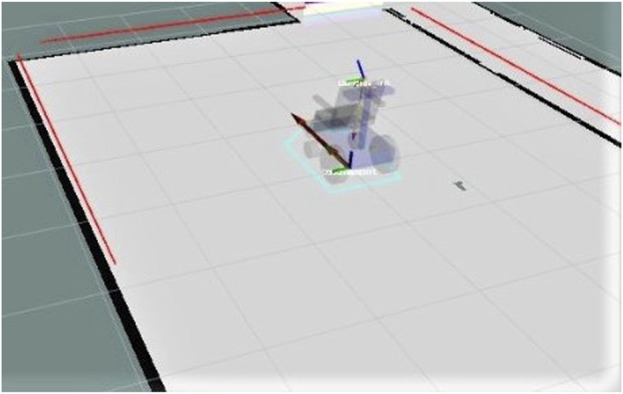
Visualization of the wheelchair robot and its environment in RViz, showing real-time sensor data, map overlays, and navigation status.

Gazebo, integrated with ROS, was used to simulate the physical environment, robot dynamics, and sensors. The mobile robot, modeled as a smart wheelchair in Figure 4, was equipped with a Hokuyo URG-04LX laser rangefinder, an IMU, and two cameras, one for localization via overhead QR codes and another front-facing camera angled downward for environment perception. Ferracuti et al. (2022).

#### 2.4.2 Virtual wall and obstacle integration

In the proposed system, virtual obstacle integration is achieved through a ROS-based architecture that allows real-time modification of the robot’s local path planning. This mechanism is essential for adapting the robot’s trajectory when new obstacles, virtual or physical, are detected or triggered by user input.

A dedicated ROS node has been developed to enable this functionality. Its role is to estimate the position of obstacles (such as virtual holes) based on the robot’s current pose and a received trigger signal. The estimated obstacle is represented as a virtual object and published as a point cloud, allowing it to be processed similarly to real sensory data by ROS navigation tools.

##### 2.4.2.1 Trigger mechanism and obstacle estimation

**FIGURE 5:**
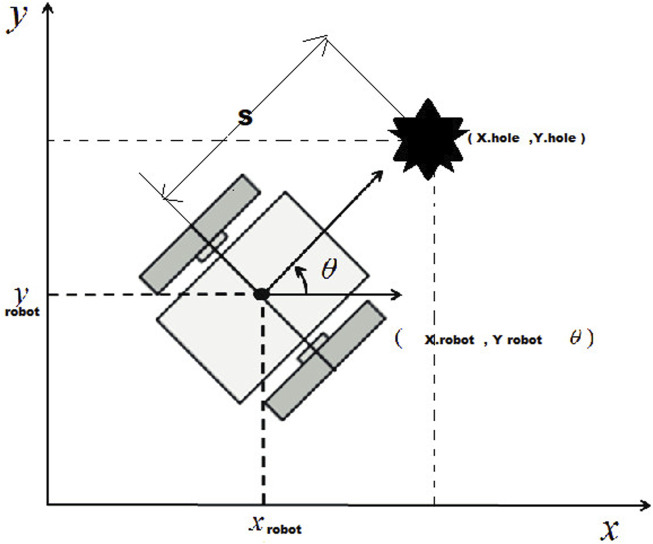
Virtual hole coordinate estimation based on robot pose and orientation.

Use the node responsible for publishing the virtual obstacle coordinates listens continuously to the robot’s odometry data and waits for a trigger message published on the ‘/trigger’ topic. When the trigger is activated (i.e., receives a value of 1), the node retrieves the current robot pose, comprising position (X,Y) and orientation θ , to estimate the coordinates of the obstacle within a 2-m range in front of the robot, as illustrated in Figure 5 and Equations 24–27. Considering the maximum robot speed of 1 m/s, gives the navigation system sufficient time to adjust the robot path and avoid any estimated obstacles triggered by the human-in-the-loop system.

The obstacle’s estimated position is computed using the following equations:

$$X_{\text{holes}}=X_{\text{robot}}+S\cdot \cos\left(\theta \right)$$ (24)

$$Y_{\text{holes}}=Y_{\text{robot}}+S\cdot \sin\left(\theta \right)$$ (25)

where $X_{\text{robot}}$ , $Y_{\text{robot}}$ , and θ are derived from the robot’s pose, and S=2 meters is the fixed distance ahead, matching the field of view of the front-facing camera.

##### 2.4.2.2 Orientation extraction from quaternions

The robot’s orientation θ is calculated from the pose’s quaternion representation:

$$\theta =2\cdot \text{atan}2\left(\sqrt{q_{i}^{2}+q_{j}^{2}+q_{k}^{2}},q_{r}\right)$$ (26)

$$q=q_{r}+q_{i}i+q_{j}j+q_{k}k$$ (27)

where $q_{r}$ , $q_{i}$ , $q_{j}$ , and $q_{k}$ represent the quaternion components describing the robot’s orientation, and i,j,k are the unit vectors of the map frame.

##### 2.4.2.3 Virtual obstacle generation and publishing

Once the coordinates of the obstacle are estimated, a virtual object—modeled as a 3D cylinder—is generated using the ROS Point Cloud Library (PCL) Rusu and Cousins (2011). This virtual point cloud is then published to a ROS topic, mimicking data from a real sensor.

This published point cloud serves two main purposes:

• Visualization: The obstacle is visualized in RViz, allowing users to confirm its position and geometry.

• Navigation Integration: The ROS navigation stack subscribes to the point cloud topic and incorporates the obstacle into the local and global costmaps, effectively altering the robot’s path to avoid it.

##### 2.4.2.4 User-defined virtual walls

A ROS-based software package was developed to enable the creation and management of virtual walls for obstacle avoidance. Through a simple user interface, operators can activate existing barriers or define new ones by entering coordinates. These are used to construct lines or splines in the X - Y plane, extruded along the Z -axis, and converted into 3D point clouds.

The generated point cloud is published as ROS sensor data, updating both local and global costmaps in the navigation stack. RViz is used for visualization and validation. Virtual wall configurations can be saved and reloaded, supporting repeatable experiments.

This system effectively simulates dynamic boundaries in scenarios where physical obstacles are impractical, offering a flexible tool for safe and adaptive navigation.

#### 2.4.3 Semantic mapping and context-aware path planning

##### 2.4.3.1 Semantic mapping from BIM files

**FIGURE 6:**
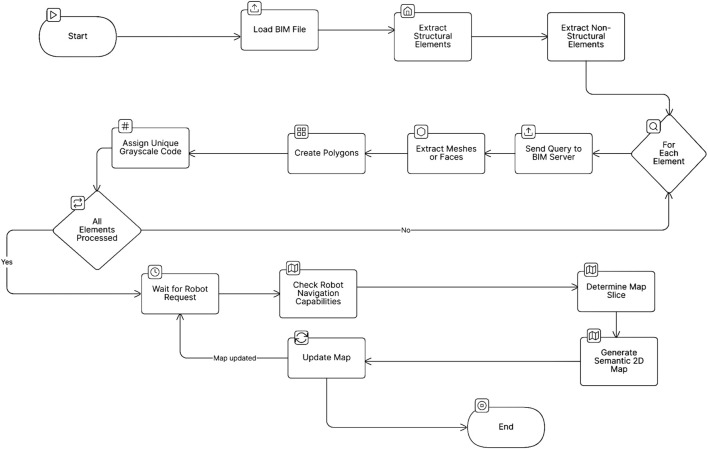
BIM-to-Robot Map Update Flow. A streamlined process illustrating the extraction of architectural data from the BIM model, semantic encoding of building elements, and generation of robot-readable maps tailored to each robot’s capabilities for accurate navigation and localization.

The used method introduces a Python-based pipeline for transforming complex 3D BIM data into structured 2D maps optimized for robotic navigation Omer et al. (2025). The process begins by selecting relevant architectural elements from the BIM model and extracting their geometric features, such as faces, edges, and vertices, at elevations aligned with the robot’s sensor height. Two primary types of maps are generated: planar localization maps and volumetric navigation maps. Each architectural component is encoded with a unique grayscale value, allowing the representation of semantic categories (e.g., walls, glass, or furniture) directly within the visual map format. This approach processes each element individually, generating its corresponding polygons and assigning class-specific grayscale values. The modular design supports both flexibility and scalability, enabling the seamless integration of new element classes by simply updating the generation scripts. As illustrated in Figure 6, the resulting map offers a semantically rich representation of the environment, enhancing robotic perception, localization, and context-aware interaction.

##### 2.4.3.2 2D semantic path planner

Semantic path planning based on A* algorithms was integrated into the ROS navigation stack, supported by Building Information Modeling (BIM) data for environmental realism Omer et al. (2024). A penalty-based cost function influenced the planner’s behavior near critical structures like walls and glass. Each map cell’s distance to the nearest wall and glass surface was precomputed and stored, allowing dynamic calculation of penalties based on proximity. This approach enables the planner to optimize paths that balance efficiency with safety and human comfort based on the user-defined functions and spacer management scenario, maintaining appropriate clearance from hazardous features.

## 3 Results and discussion

**FIGURE 7:**
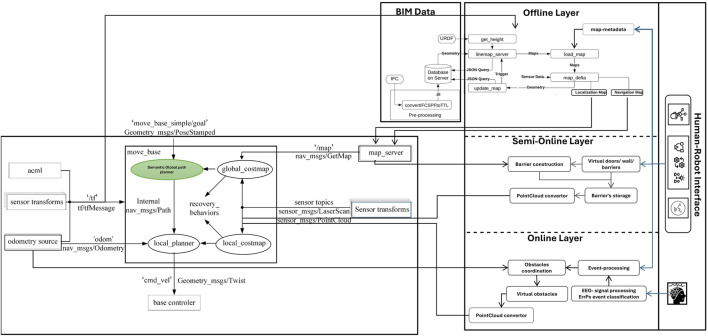
Schematic of all robot ROS packages and their integration with the three control layers: online, semi-online, and offline controllers. These layers work together to override the robot’s behavior, ensuring safety and compliance with user requirements.

The simulation results are categorized into three key aspects according to the controller layers, as illustrated in Figure 7.

### 3.1 Online layer simulation

**FIGURE 8:**
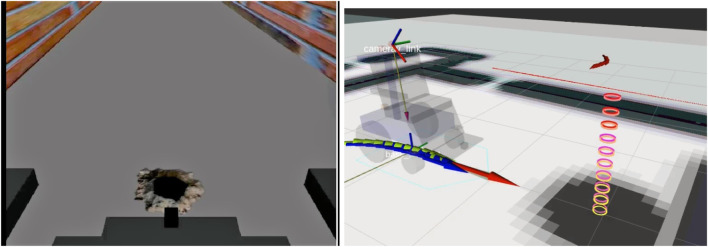
Left: The user perceives obstacles that the robot cannot detect. Right: Upon user trigger, the online layer sends virtual obstacles to the robot, allowing it to adjust its navigation path accordingly.

The online layer simulation focuses on the generation of virtual obstacles based on user input. When the user detects an obstacle that is not captured by the robot’s onboard sensors, a trigger is sent. These results were obtained from data collected during the robot’s navigation, where users were seated in front of a screen watching the robot’s simulation in real time, as shown in Figure 8. Users were instructed to press a keyboard key whenever they identified an obstacle centered in the robot’s camera view that was not detected by the onboard sensors. Upon receiving this trigger, the system generated a virtual obstacle and placed it onto the robot’s virtual map at coordinates estimated using Equations 24–27.

The analysis of all 82 trials provides valuable insights into the dataset’s performance and reliability, which are summarized in Table 1. The average measurement was calculated as 0.246 m, representing the central tendency of the trials. The minimum and maximum values observed were 0.02 m and 0.75 m, respectively, showcasing the range of variability within the data. The variability around the mean was characterized by a standard deviation of 0.148 m and a variance of 0.0219 m^2^;, indicating a moderate spread in measurements.

**TABLE 1:** Statistical parameters from 82 trials evaluating virtual obstacle coordinate estimation, detection, and avoidance accuracy.

Parameter	Value	Unit
Average	0.246	m
Minimum (Min)	0.02	m
Maximum (Max)	0.75	m
Standard Deviation	0.148	m
Variance	0.0219	m^2^
Avoidance accuracy	96	%

Furthermore, based on the acceptable error threshold of 0.5 m, which accounts for the inflation area around the robot’s footprint, the data shows that any value below this threshold allows the robot to avoid obstacles successfully. With only three trials exceeding this threshold, the accuracy of the trials was calculated to be approximately 96%.

### 3.2 Semi-online layer simulation

**FIGURE 9:**
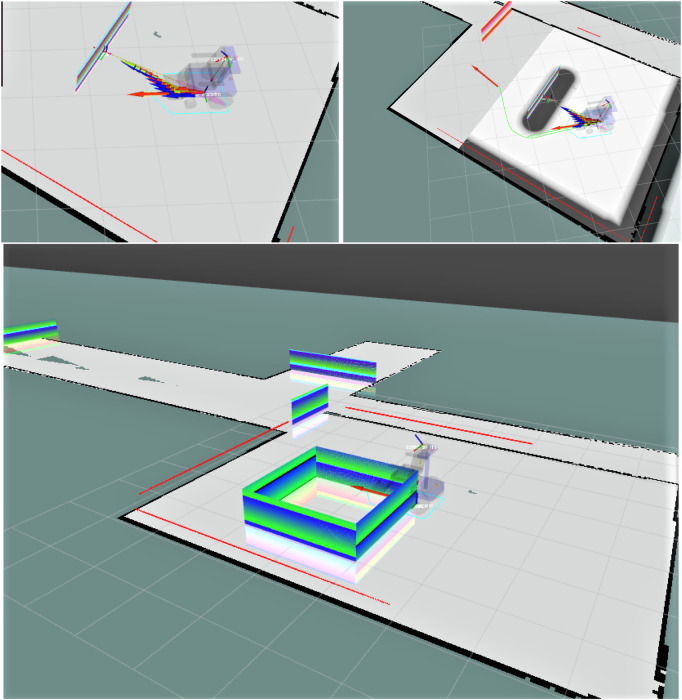
Testing different types of visual borders and walls using the SIM-Online Layer Controller to observe how the robot interacts with virtual barriers.

The second set of results explores the use of virtual borders and barriers to restrict access to specific areas. These barriers, defined by the user through a graphical interface, are used to block certain zones, such as doorways or entire sections of a map, as can be seen in Figure 9. Once defined, the virtual boundaries are translated into 3D point cloud data and integrated into the ROS navigation stack. This ensures that the robot’s path planning process respects these artificial constraints, enabling flexible environmental control during navigation tasks.

The computational cost of implementing virtual barriers as 3D point clouds was analyzed for scenarios in a smart home environment. Standard indoor door dimensions were utilized for calculations, with each point spaced 1 cm apart, aligning with the resolution commonly applied in robotics for obstacle detection and navigation.

In the worst-case scenario, a smart home containing five rooms and two additional restricted areas was considered. The estimated number of points required to represent virtual barriers varied depending on the publishing rate, with two cases analyzed: 1 Hz and 5 Hz. The computational costs for different scenarios are summarized in Table 2.

**TABLE 2:** Computational cost for virtual barriers with standard door dimensions.

Scenario	Points at 1 Hz	Total points sent at 1 Hz	Total points sent at 5 Hz
One Standard Door	8,000	8,000	40,000
Seven Standard Doors	56,000	56,000	280,000
Square Barrier ( 2×2 meters) with Standard Door Height	240,000	240,000	1,200,000

Modern commercial computers, equipped with multi-core CPUs, substantial RAM (8–32 GB), and high-speed networking, are well-suited to handle these computational demands. Additionally, single-board computers such as the Raspberry Pi 4 Model B—with its quad-core processor, RAM configurations (2 GB, 4 GB, or 8 GB), and gigabit networking capabilities—demonstrate sufficient processing power to manage the generation, handling, and transmission of point cloud data even in the worst-case scenario.

### 3.3 Offline layer simulation

**FIGURE 10:**
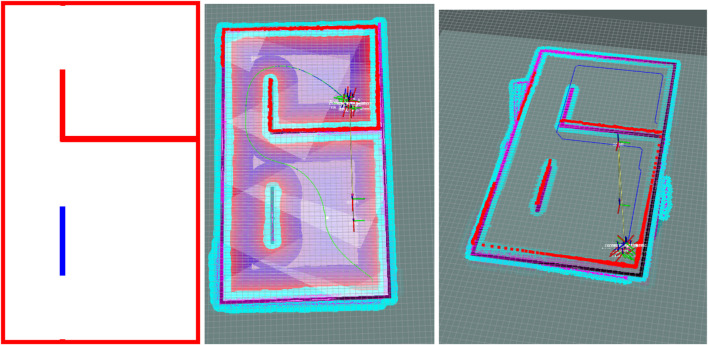
Left: 3D model of the simulated environment alongside its corresponding projected map, where walls and glass surfaces are depicted, with glass surfaces highlighted in blue. Midel: Comparison between conventional A* path planning and semantic A* path planning under user-specified constraints, where the robot is instructed to maintain a safe distance from glass surfaces while staying close to walls. Right: Path generated using conventional A*; Left: Path generated using semantic A*.

Finally, we demonstrate the implementation of user-specific navigation rules within a controlled workspace environment. In this case study in Figure 10, the robot is tasked with maintaining a safe distance from glass partitions, addressing critical safety considerations. These navigation rules are encoded in metadata as configuration parameters, defining relevant objects and spaces within the environment, as well as the desired interaction behaviors.

Each robot is assigned a unique configuration file that specifies its authorized behaviors and governs its navigation strategies. Simulation results indicate that the path planner reliably complies with these constraints, generating trajectories that consistently maintain safe distances from hazardous structures. This underscores the system’s flexibility and its capacity to incorporate individualized user preferences into autonomous decision-making processes.

## 4 Conclusion

This study presents a significant advancement in the development of autonomous navigation systems for mobile robots, with a focus on supporting vulnerable groups. By implementing a robust three-layer control framework comprising “online”, “semi-online”, and “offline” approaches, as illustrated in Figure 2, new navigation strategies were investigated. These strategies are designed to enhance safety and efficiency while minimizing reliance on complex sensor systems and computationally intensive resources.

The core concepts of this work emphasize improving mobile robot navigation within a human-centered design framework. This enhancement is achieved through direct interventions, such as correcting the robot’s behavior when its sensors fail to detect obstacles, a technique applied in the first (“online”) layer. Nevertheless, the “online” layer has limitations, as obstacle coordinates are estimated only within a 2-m range, assuming that the human observer has a line of sight aligned with the robot’s current pose. In our current implementation, we used a fixed camera mounted at the front of the robot with a focal range of 2 m, ensuring alignment under controlled conditions. However, if the user is not actively observing the screen or the line of sight is obstructed, it becomes difficult to ensure accurate human intervention.

Furthermore, risks are mitigated through partial or complete access restrictions in hazardous zones, as addressed by the subsequent (“semi-online” and “offline”) layers. The “semi-online” layer sets time- and area-specific restrictions, while the “offline” layer establishes permanent navigation rules based on the semantic understanding of different zones and their associated equipment. These rules remain valid as long as the user’s requirements remain unchanged. A key limitation of the “semi-online” layer is that it is still manually operated and dependent on human intervention. This layer could be significantly enhanced by integrating data from other autonomous systems. For example, if it could detect scheduled activities such as floor cleaning, it could automatically trigger virtual barriers or close doors. Moreover, integration with IoT sensors would allow the system to detect special events and small objects, such as pets, kids, or toys, and then set the virtual barriers to block that area without requiring human input.

The “offline” control layer introduces a key innovation by implementing permanent rule-based strategies derived from a semantic understanding of the environment. Leveraging Building Information Models (BIMs) integrated with digital twins, this layer efficiently utilizes architectural data to optimize robot navigation. A critical advancement in this layer is the direct generation of robot-specific navigation maps from BIM data. This process significantly reduces computational resource usage compared to traditional methods like SLAM or LiDAR, generating maps in under a second Peavy et al. (2023); Naik et al. (2019). This efficiency is particularly advantageous for large structures, simplifying the mapping process while ensuring high accuracy.

Additionally, the generated maps are aligned with each robot’s sensory capabilities, improving localization and navigation accuracy. This ensures that obstacles often overlooked by conventional sensors, such as transparent surfaces or furniture with thin legs, are effectively represented in the maps Liu et al. (2024); Trautmann et al. (2023). Such adaptations enhance the robot’s ability to navigate diverse environments safely and efficiently.

The “offline” layer also incorporates semantic path planning, transforming operational rules and user requirements into global paths that account for both geometrical and contextual constraints. Robots can prioritize or avoid certain areas based on the presence of vulnerable individuals, the specific function of zones, or operational restrictions Loganathan and Ahmad (2023). By tailoring navigation paths to align with the environment’s intended use, the “offline” layer improves both safety and operational efficiency. However, the current approach assumes that all buildings have accurate and up-to-date BIM files and that existing furniture coordinates are already encoded. While this enables efficient map generation, it limits applicability in unmodeled or dynamically changing environments. The system would be more robust if it included the ability to update furniture layouts or even generate BIM files from scratch. This could be achieved by deploying more capable robots equipped with advanced scanning capabilities to autonomously construct BIM models, which can then be used by simpler robots with limited navigation skills.

Ultimately, this study highlights the importance of integrating human-centered control with full autonomy in mobile robot navigation systems. The findings demonstrate substantial improvements in computational efficiency, adaptability, and obstacle avoidance. The proposed framework bridges the gap between manual control and autonomy, enabling robots to adapt to dynamic, user-defined environments. This flexibility is crucial for assistive technologies, where robots can be personalized to navigate complex spaces safely and provide greater independence for vulnerable individuals. This hybrid approach, combining autonomy with human-centered interventions, ensures that robots can operate effectively while maintaining sensitivity to user needs, making them an ideal solution for a wide range of assistive applications.

## Data Availability

The original contributions presented in the study are included in the article/supplementary material, further inquiries can be directed to the corresponding author.
